# Optimal administration strategies of tranexamic acid to minimize blood loss during spinal surgery: results of a Bayesian network meta-analysis

**DOI:** 10.1080/07853890.2022.2101687

**Published:** 2022-07-21

**Authors:** Ziqin Cao, Qiangxiang Li, Jia Guo, Yajia Li, Jianhuang Wu

**Affiliations:** a Department of Spine Surgery and Orthopaedics, Xiangya Hospital, Central South University, Changsha, P. R. China; b Ningxia Geriatric Disease Clinical Research Center, People’s Hospital of Ningxia Hui Autonomous Region, Yinchuan, P. R. China; c National Clinical Research Center for Geriatric Disorders of Xiangya Hospital, Central South University (Sub-Center of Ningxia), Yinchuan, P. R. China; d Department of Hunan Institute of Geriatrics, Hunan People’s Hospital, Changsha, P. R. China; e Department of Dermatology, Xiangya Hospital, Central South University, Changsha, P. R. China; f National Clinical Research Center for Geriatric Disorders, Xiangya Hospital, Central South University, Changsha, P. R. China

**Keywords:** Tranexamic acid, spinal surgery, blood conservation, network meta-analysis, comparative efficacy and safety

## Abstract

**Background:**

Tranexamic acid (TXA) has been widely used for bleeding reduction in spinal surgery. Available evidence is insufficient to inform clinical decisions making and there remains a lack of comprehensive comparisons of dose regimens and delivery routes. This study is aimed to assess and compare different strategies regarding the involvement of TXA in spinal surgery for the optimal pathway of efficacy and safety.

**Materials and methods:**

Cochrane Library, PubMed, Embase, Scopus and CNKI were searched for the period from January 1990 to October 2021. A random-effect model was built in the Bayesian network meta-analysis. The surface under the cumulative ranking analysis (SUCRA) and clustering rank analysis was performed for ranking the effects.

**Results:**

The current network meta-analysis incorporated data from 33 studies with 3302 patients. Combination administration showed superior effects on reducing intraoperative bleeding (SUCRA 78.78%, MD −129.67, 95% CI [(−222.33, −40.58)]) than placebo, and was ranked as top in reducing postoperative bleeding (SUCRA 86.91%, MD −169.92, 95% CI [(−262.71, −83.52)]), changes in haemoglobin (SUCRA 97.21%, MD −1.28, 95% CI [(−1.84, −0.73)]), and perioperative blood transfusion (SUCRA 93.23%, RR 0.10, 95% CI [(0.03, 0.25)]) simultaneously, and was shown as the best effectiveness and safety (cluster-rank value for IBL and VTE: 4057.99 and for TRF and VTE: 4802.26).

**Conclusions:**

Intravenous (IV) plus topical administration of TXA appears optimal in the reduction of perioperative bleeding and blood transfusion, while the local infiltration administration is not effective for blood conservation. Further studies are required to verify the current findings.

## Introduction

Surgery is the only effective option for some end-stage degenerative spinal diseases, such as unstable vertebral fracture, scoliosis and spondylolisthesis. However, the significant perioperative blood loss associated with such procedures leads to an increased risk of complications, including postoperative hypotension, acute anaemia and infection [1,2]. Blood loss also increases the necessity for blood transfusions which incur other side effects, including transmission of infectious diseases, haemolytic reaction, postoperative epidural haematoma of the spinal cord, allergic transfusion reaction and impose an economic burden on health services [3].

Tranexamic acid (TXA) is an antifibrinolytic drug, acknowledged to reduce blood loss in knee arthroplasty, heart surgery, craniosynostosis and extensive spinal surgery [4–7]. Normally, proteins involved in the fibrinolytic pathway interact via lysine-containing binding sites, leading to their proteolysis by serine proteases. TXA is a lysine analogue which competitively inhibits tissue plasminogen activator (TPA), plasminogen (plasmin precursor), and plasmin activity by blocking the lysine binding site. By this action, TXA reduces platelet degradation, promotes thrombosis and reduces bleeding during an operation [8]. It can be administered via an intravenous (IV), oral or local route, although the IV route is the most favoured, according to the literature [9,10]. A usual loading dose of 10–15 mg/kg may be continuously titrated during the operation with the minimum effective dose of 15 mg/kg being previously reported [11]. A dose as high as 50 mg/kg has previously been used during spine and skull surgery with no serious complications ensuing [12–14].

Numerous systematic reviews have been published regarding the use of TXA for spinal surgery but reports have been highly heterogeneous. Previous reviews and meta-analyses have reported a reduction in blood loss in a variety of spinal procedures, including cervical laminoplasty, posterior lumbar interbody fusion, adult spinal deformity correction and posterior spinal joint fusion. However, these studies analysed data from a variety of procedures with each study involving different dosing methods, dosages and single/combination approaches, making optimal strategies for spinal surgery difficult to identify. This study is a network meta-analysis for the assessment of different strategies regarding the involvement of TXA in spinal surgery, including efficacy and safety.

## Method

### Literature searches and study selection

The study was guided by the Preferred Reporting Items for Systematic Reviews and Meta-Analyses (PRISMA) guidelines [15]. The current network meta-analysis was registered on the PROSPERO database (registration number: CRD42022296855). Two authors (JG and JHW) conducted independent searches of Cochrane Library, PubMed, Embase, Scopus and CNKI for literature with a publication date range from January 1990 to October 2021. The following search terms were utilized: “spine” OR “spinal surgery” OR “vertebral” OR “vertebral surgery” OR “cervical” OR “cervical surgery” OR “thoracic” OR “thoracic surgery” OR “lumbar” OR “lumbar surgery” AND “antifibrinolytic” OR “tranexamic acid” OR “TXA”. A secondary search was performed in the reference lists of eligible articles. There was no language restriction placed for publications. A research protocol that followed the PICO principle was pre-drafted: 1. Population: patients who underwent spinal surgery; 2. Intervention: perioperative use of TXA; 3. Comparison: different TXA administration strategies; 4. Outcomes: intra- and post-operative blood loss, changes in haemoglobin in the 24-h postoperative period, perioperative blood transfusion rate and incidence of venous thrombosis (VTE).

By the PICO protocol, studies with the following characteristics were included [1]: patients undergoing spinal surgery [2]; perioperative use of TXA to reduce blood loss [3]; comparison of ≧2 types of TXA administration strategies (intercomparison or compared to placebo) [4]; RCTs of prospective design with parallel groups were utilized and [5] the primary outcome of intraoperative blood loss (IBL) was reported.

Exclusion criteria were as follows [1]: involvement of surgery other than spinal [2]; single-arm design [3]; study protocols, animal research, *in vitro* basic research studies, observational research, reviews or systematic reviews, conference paper and letter to the editor.

Corresponding authors were contacted where studies did not report original data. We would exclude the study when receiving no reply from corresponding authors. Corresponding authors were also contacted where data were only presented in the form of figures. When receiving no reply, two authors made independent attempts to extract the data from the figures, and those studies with unextractable data in figures were still excluded. All differences were settled by discussion.

### Data extraction and quality assessment

Study quality was assessed by two authors (QXL and JG) under the guidance of the Cochrane risk of bias assessment tool [16]. There were six items for evaluation of bias risk (sequence generation, allocation concealment, blinding, incomplete outcome data, selection outcome reporting, and other sources of bias) and three ranking levels (low, unclear and high) were included. Where one or more high-risk items occurred within a study, it was rated as global high risk and excluded.

Two authors (ZQC and YJL) performed independent extraction of information from eligible studies, which included first author, year of publication, number of participants, mean age, gender, mean BMI (body mass index), disease diagnosis, the surgery type and outcome data. The authors gave priority to the data obtained with intention to treat analysis to minimize the impact of withdrawal bias.

### Outcome measurement

The primary efficacy endpoint was IBL. Postoperative blood loss (PBL), change in haemoglobin during the 24-h postoperative period (HBC) and perioperative blood transfusion rate (TRF) was chosen as the secondary efficacy endpoints due to their importance in clinical decision making. Differences between 24 h postoperative timepoint and baseline values of HBC were used to evaluate efficacy to minimize any bias due to differences in baseline values. Operative blood loss is presented as a weighted mean difference (WMD) with a 95% confidence interval (CI). In those studies in which no change from baseline values was reported, the correlation coefficient method was performed due to the recommendation of the Cochrane Handbook [17]. The safety endpoint was the incidence of postoperative VTE, including symptomatic or non-symptomatic DVT and PE, and is presented as the risk ratio (RR) with a 95% CI.

### Statistical analysis

A random-effects network meta-analysis using a Bayesian framework was conducted by R software version 4.1.2 (R Foundation for Statistical Computing, Vienna, Austria) with GeMTC (version 1.0 − 1) and JAGS packages (version 4.3.0, https://sourceforge.net/projects/mcmc-jags/). The Markov-chain Monte Carlo (MCMC) method was used to obtain the non-informative uniform and normal prior distributions and the convergence of iterations was assessed by Gelman − Rubin − Brooks statistic [18,19]. Four iteration chains, with 20,000 iterations per chain, were set to fit the model and calculate the posterior distributions of model parameters. The thinning interval was set at 10 and the burn-ins at 1000 for each chain. MD and RR with 95% CI were generated from the posterior distribution medians. Of 95% CI did not contain 1 for RRs or 0 for MDs indicating significant differences between interventions. A value of *p* < .05 was considered statistically significant. The surface under the cumulative ranking value (SUCRA) derived from posterior probabilities was used to rank the relative efficacy and safety of interventions with higher SUCRA values indicating better interventions [20]. Clustered ranking plots were used for the determination of optimal intervention choice.

Global heterogeneity of each endpoint model was evaluated by I2 tests: <25% indicated low heterogeneity while >50% indicated high heterogeneity [21]. Deviance information criterion (DIC) was obtained from consistency and inconsistency models for each endpoint and differences between each pair of DICs (dDIC) were calculated to assess global inconsistency. A value of dDIC >10 indicated appreciable global inconsistency. A node-split model was used to check local inconsistency for each endpoint with a *p*<.05 indicating significant local inconsistency. Sensitivity analysis was conducted to identify any sources of inconsistency. Publication bias within each network was evaluated using a funnel plot and Egger’s test. The occurrence of asymmetry in a network funnel plot or a *p* value <.05 for Egger’s test indicated significant publication bias. A network meta-regression analysis was performed using R software with the Gemtc package to evaluate the potential impact of confounding factors on the model based on non-negligible differences in participant baseline characteristics [22].

## Results

### Literature selection and study characteristics

**Figure 1.**
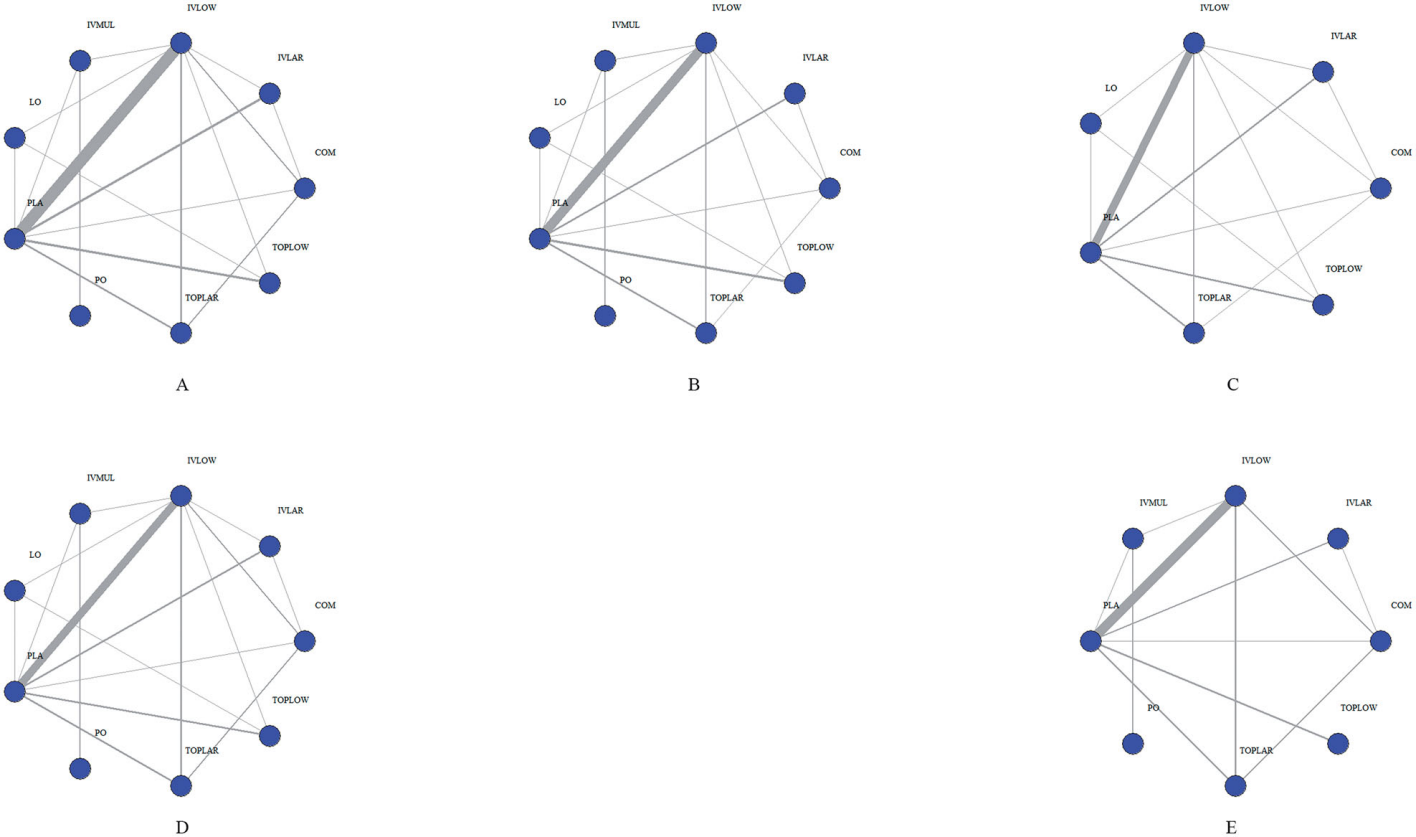
Structure of network formed by interventions. The lines between treatment nodes indicate the direct comparisons made within randomized controlled trials. (A) IBL. (B) PBL. (C) HBC. (D) TRF. (E) VTE. IBL: intraoperative blood loss; PBL: postoperative blood loss; HBC: change in haemoglobin during the 24-h postoperative period; TRF: perioperative blood transfusion rate; VTE: venous thrombosis.

A total of 33 studies [6,23–53] involving 26 different TXA administration strategies were included (Supplementary Figure 1). The 26 strategies were divided into nine groups based on AAHKS/AAOS/ASRA/AKS/AHS guidelines [54] on the use of TXA for arthroplasty: low-dose IV (<20 mg/kg or ≤1 g, IVLOW), high-dose IV (≥20 mg/kg or > 1 g, IVLAR), low-dose topical (≤1.5 g, TOPLOW), high-dose topical (>1.5 g, TOPLAR), combined use (IV plus topical, COM), oral (PO), local infiltration (equivalent bilateral administration of TXA into paraspinal muscles prior to incision, LO), multiple IV use (multiple IV infusion before and after surgery, IVMUL) and placebo (PLA). Network plots are presented in Figure 1.

The current network meta-analysis enrolled a total of 3302 patients. The median age across all studies was 51.75 years (interquartile: 45.54 − 55.60) and the median percentage of male patients was 45.49% (interquartile: 31.97 − 56.79%). Three studies did not report patients’ diagnoses, five studies did not restrict diagnoses and most studies (12 of 33) focussed on degenerative spinal diseases (Supplementary Table 1). Supplementary material shows the quality and bias-risk assessments (Supplementary Table 2), funnel plots (Supplementary Figure 2), Egger’s tests (Supplementary Figure 3) and results of global and local inconsistency tests (Supplementary Table 3 and Supplementary Figure 4).

The following four covariates were selected for network meta-regression after consultation with spine surgery experts and a review of the literature: publication year, mean BMI, mean age and disease type. Supplementary Table 4 presents the detailed results of Bayesian network meta-regression analyses. Results of model fit and iteration convergence assessments are also shown (Supplementary Figures 5 and 6).

### Intraoperative blood loss

The IBL network included 33 studies with 3302 patients. Model fit and iteration convergence were both good. A dDIC value of 1.27 (75 data points) and *p* value >.05 indicated no significant inconsistency. A significant interaction between IBL and diagnoses and surgery types of participants (*β* 113.94, 95% CI [(32.27, 190.86)]) was shown by network meta-regression. No publication bias or evidence of high heterogeneity among trials (*I*^2^ = 0.9%) was found.

SUCRA rankings identified IVLAR as the most likely effective strategy for reducing IBL (SUCRA 81.59%), followed by COM (SUCRA 78.78%) and IVLOW (SUCRA 69.98%). TOPLAR had the lowest ranking (SUCRA 26.64%). IVLAR (MD −137.63, 95% CI [(−234.29, −47.84)]), COM (MD −129.67, 95% CI [(−222.33, −40.58)]) and IVLOW (MD −108.6, 95% CI[(−151.51, −68.65)]) were all significantly superior to PLA.

### Postoperative blood loss

The PBL network included 26 studies with 2576 patients. The network showed a good model fit and iteration convergence with no global inconsistency (dDIC = 0.06, 75 data points). However, a distinct local inconsistency between COM and PLA was shown by node-split tests (*p* value = .03814). Network meta-regression showed no association among PBL and publication year, mean age, mean BMI or disease type and no significant heterogeneity (*I*^2^ = 0%). However, a publication bias was reported by Egger’s test (*p* value = .049).

SUCRA rankings demonstrated that COM was most likely to be the most effective strategy for reducing PBL (SUCRA 86.91%), followed by PO (SUCRA 62.04%) and IVMUL (SUCRA 60.42%) while LO ranked the lowest (SUCRA 16.58%). PO (MD −127.83, 95% CI [(−307.13, 52.96)]) and LO (MD −20.95, 95% CI [(−136.96, 93.12)]) showed no significant difference compared with PLA.

### Changes in haemoglobin

The HBC network included 20 studies with 1920 patients. A good model fit and iteration convergence were indicated with no inconsistencies from the global consistency (dDIC = 0.85, 49 data points) or node-split tests (*p* value >.05). Network meta-regression indicated no association between HBC and publication year, mean age, mean BMI or disease type, and no significant heterogeneity (*I*^2^ = 4%). No publication bias was evident. Other seven groups except PO and IVMUL were included in this network.

Of the seven groups analysed in this network, all were significantly superior to PLA in reducing HBC except LO. SUCRA rankings put COM at the top (SUCRA 97.21%, MD −1.28, 95% CI [(−1.84, −0.73)]), followed by TOPLOW (SUCRA 82.15%, MD −0.93 94% CI [(−1.36, −0.48)]) and IVLAR (SUCRA 56.23%, MD −0.57, 95% CI [(−0.97, −0.18)]). LO (SUCRA 13.98%, MD −0.04, 95% CI [(−0.66, 0.58)]) occupied the lowest ranking.

### Perioperative blood transfusion

The TRF network incorporated 24 studies with 2560 patients. Model fit and iteration convergence were both good, and there showed no inconsistencies using either the global consistency (dDIC = 5.90201, 57 data points) or node-split tests (*p* value > .05). No publication bias was detected. A significant interaction between TRF and mean BMI was indicated by network meta-regression (*β* 1.52, 95% CI [(0.34, 2.76)]). No significant heterogeneity among the trials was found (*I*^2^ = 0%).

All routes of administration were significantly superior to PLA in reducing TRF except IVMUL (RR 0.32, 95% CI [(0.07, 1.05)]) and LO (RR 0.72, 95% CI [(0.25, 1.80)]). SUCRA rankings put COM first (SUCRA 93.23%), followed by PO (SUCRA 82.21%) and IVLAR (SUCRA 74.79%) while LO occupied the lowest ranking (SUCRA 22.05%).

### Incidence of VTE

The VTE network incorporated 26 studies with 2624 patients. Model fit and iteration convergence were both good and no inconsistencies were detected either globally (dDIC = 1.98195, 59 data points) or from node-split tests (*p* value >.05). No publication bias was reported. Network meta-regression showed no association among VTE and publication year, mean age, mean BMI or disease type, and no significant heterogeneity (*I*^2^ = 4%).

The safety network included 8 groups but not LO. No significant differences were found when compared with PLA. SUCRA rankings showed that TOPLOW was most likely to be the safest route (SUCRA 82.56%), followed by IVLOW (SUCRA 52.99%) and COM (SUCRA 51.51%). TOPLAR (SUCRA 31.11%) ranked lowest.

**Figure 2.**
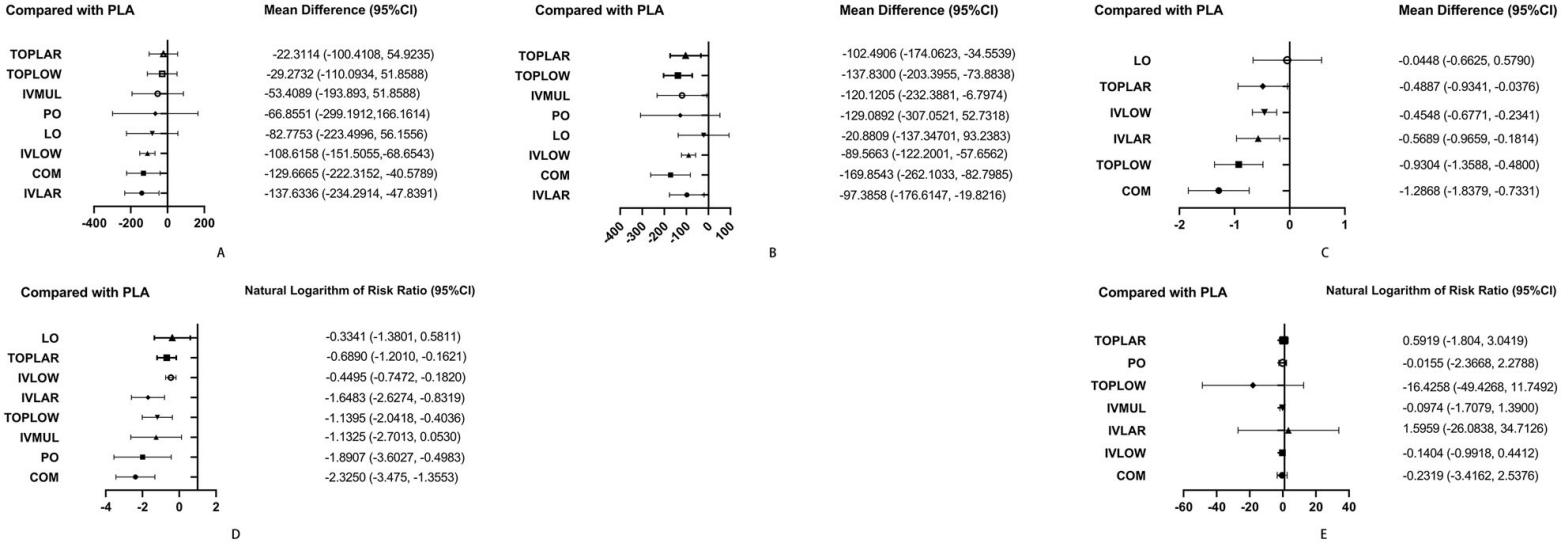
Forest plots. (A) IBL. (B) PBL. (C) HBC. (D) TRF. (E) VTE. (The results of TRF and VTE are expressed as the natural logarithm of risk ratio. Differences between treatments were considered significant when the 95% CI did not contain 0 for lnRRs and MDs). RR: risk ratio; MD: weighted mean difference; IBL: intraoperative blood loss; PBL: postoperative blood loss; HBC: change in haemoglobin during the 24-h postoperative period; TRF: perioperative blood transfusion rate; VTE: venous thrombosis.

**Figure 3.**
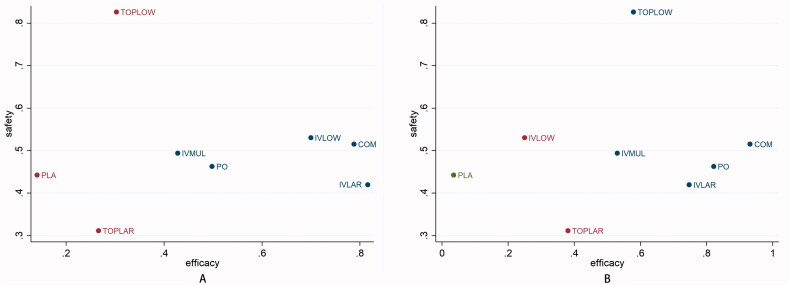
Cluster-rank plots. (A) The cluster-rank plot of IBL and VTE. (B) The cluster-rank plot of TRF and VTE. (The cluster-rank value is the product of the abscissa and ordinate of each treatment). IBL: intraoperative blood loss; TRF: perioperative blood transfusion rate; VTE: venous thrombosis.

Detailed SUCRA results are shown in Table 1 and Supplementary Figure 7. Forest plots are shown in Figure 2. Relative efficacies and safeties of all treatments (league plots) are summarized in Tables 2 and 3. Cluster-rank results (Figure 3) revealed COM to have the greatest potential to be the optimum strategy (cluster-rank value for IBL and VTE: 4057.99 and for TRF and VTE: 4802.26).

**Table 1.** Detailed results of main analysis.

Treatment	MD (95% CI) for IBL	SURCA for IBL, %	MD (95% CI) for PBL	SURCA for PBL, %	MD (95% CI) for HBC	SURCA for HBC, %	ln RR (95% CI) for TRF	SURCA for TRF, %	ln RR (95% CI) for VTE	SURCA for VTE, %
PLA	Reference	14.08	Reference	5.85	Reference	7.68	Reference	3.57	Reference	44.22
LO	−82.78 (−223.50, 56.16)	55.99	−20.80 (−137.60, 92.71)	16.58	−0.04 (−0.66, 0.58)	13.98	−0.35 (−1.37, 0.58)	22.05	NA	NA
PO	−66.86 (−299.19, 166.16)	49.82	−127.19 (−307.74, 54.15)	62.04	NA	NA	−1.90 (−3.69, −0.48)*	82.21	−0.02 (−2.37, 2.28)	46.26
TOPLOW	−29.27 (−110.09, 51.86)	69.98	−137.53 (-203.76, −73.66)*	73.70	−0.93 (−1.36, −0.48)*	82.15	−1.15 (−2.01, −0.40)*	57.93	−16.43 (−49.43, 11.75)	82.56
TOPLAR	−22.31 (−100.41, 54.92)	26.64	−102.35 (−173.52, −34.87)*	52.40	−0.49 (−0.93, −0.04)*	48.32	−0.68 (−1.20, −0.17)*	38.12	0.59 (−1.80, 3.04)	31.11
IVLOW	−108.62 (−151.51, −68.65)*	30.30	−89.50 (−122.26, −57.58)*	42.73	−0.45 (-0.68, −0.23)*	44.43	−0.45 (−0.75, −0.18)*	25.05	−0.14 (−0.99, 0.44)	53.00
IVLAR	−137.63 (−234.29, −47.84)*	81.59	−97.48 (−176.57, −20.15)*	49.36	−0.57 (−0.97, −0.18)*	56.24	−1.66 (−2.66, −0.83)*	74.79	1.60 (−26.08, 34.71)	41.94
IVMUL	−53.41 (−193.89, 86.05)	42.81	−118.55 (−229.25, −7.66)*	60.42	NA	NA	−1.14 (−2.79, 0.07)	53.05	−0.10 (−1.71, 1.39)	49.40
COM	−129.67 (−222.32, −40.58)*	78.78	−169.92 (−262.71, −83.52)*	86.92	−1.29 (−1.84, −0.73)*	97.21	−2.34 (−3.47, −1.38)*	93.23	−0.23 (−3.42, 2.54)	51.51

The results of TRF and VTE are expressed as the natural logarithm of risk ratio.

*Significant difference compared to placebo.

NA: not applicable; IBL: intraoperative blood loss; PBL: postoperative blood loss; HBC: change in haemoglobin during the 24-h postoperative period; TRF: perioperative blood transfusion rate; VTE: venous thrombosis; PLA: placebo; LO: local infiltration; PO: oral; TOPLOW: low-dose topical; TOPLAR: high-dose topical; IVLOW: low-dose intravenous; IVLAR: high-dose intravenous; IVMUL: multiple intravenous; COM: combined use (intravenous plus topical); WMD: weighted mean difference; SUCRA: the surface under the cumulative ranking analysis

**Table 2.**
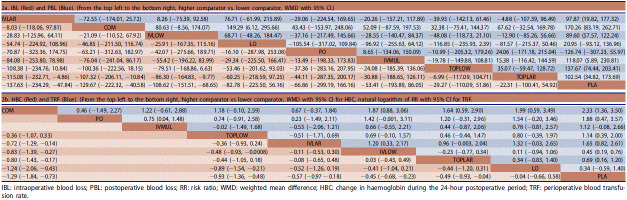
The league plots of efficacy endpoints.

**Table 3.**
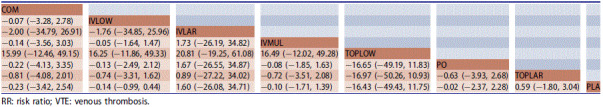
The league plots of safety endpoint. VTE (Red. (From the top left to the bottom right, higher comparator vs. lower comparator, natural logarithm of RR with 95% CI.).

## Discussion

TXA has been widely used during spinal and other orthopaedic surgeries and numerous studies have reported its safety and effectiveness in reducing perioperative blood loss. Clinical guidelines have been devised to normalize the use of TXA during general surgery but there is little guidance specific to spinal surgery. A systematic review by Yerneni et al. indicated a moderate reduction in PBL by topical use of TXA [14]. Xiong et al. [55] concluded that both high and low-dose IV TXA were effective and safe during surgery for adolescent spinal deformity. However, the available evidence is insufficient to inform clinical decisions making and there remains a lack of comprehensive comparisons of dose regimens and delivery routes.

The current network meta-analysis is the first to synthesize all relevant, high-quality RCTs to comprehensively compare the efficacy and safety of different administration routes for TXA during spinal surgery. The main findings may be summarized as follows: (1) SUCRA rankings showed that IV TXA administration may have a superior effect on reducing IBL to topical, oral or local infiltration strategies, although no significant difference was found between IV and other strategies; (2) SUCRA rankings showed that topical TXA application was more effective in reducing PBL than IV infusion, although, similarly, there was no significant difference between topical and IV routes; (3) Local infiltration of TXA performed worst, with only a small or without significant reduction in IBL, PBL, HBC and TRF compared with placebo; (4) All TXA administration routes were well-tolerated and safe compared with placebo; (5) Network meta-regression demonstrated that BMI may affect perioperative blood transfusion rate and the nature of the surgical operation may affect intraoperative bleeding. This result is consistent with the findings of Jiang et al. [56] that obesity was associated with greater blood loss during spinal surgery; (6) Cluster rank analysis indicated that a combination of IV and topical use could be the optimum administration strategy for blood conservation during spinal surgery.

In the role of haemostasis in surgery, IV, oral and local administration of TXA is selected. In previous studies related to surgery, IV administration is the most common [9,10]. A meta-analysis (6 studies, 411 patients) by Brown et al. [57] revealed that the most common route of administration in laminectomy and fusion with posterior instrumentation was a preoperative IV injection of 15 mg/kg TXA, and the blood loss of patients using TXA was significantly reduced. In addition, when the loading dose was as high as 50 mg/kg, with no significant complications in spine and knee surgery before [12–14]. This was also consistent with our findings that no significant increased safety risk was found for TXA even in the high-dose group.

TXA was proved to be safe and effective in reducing surgical bleeding in spine surgery, but there was still no conclusion on the optimal application scheme. Heterogeneity in the definition of high and low doses in previous studies also reduced the validity of their results. At the same time, there were many complex routes of TXA administration, and TXA administration strategies and their definitions were sometimes unclear in actual studies. Therefore, the results of reviews and meta-analyses of TXA use strategies in spine surgery should be carefully interpreted, especially when recommendations for high-dose TXA were proposed based on the meta-analysis results [14,58]. It was important to understand that the optimal dose regimen for one route may not be optimal for another route. Therefore, we summarized the experience of the former, defined different dose groups and routes of administration based on existing guidelines, and conducted rigorous statistical methods including SUCRA and Cluster-rank by using mesh meta-analysis of the Bayesian framework to comprehensively analyse this problem. We believe that the results of this network meta-analysis will be useful for clinical decision making.

There are also some limitations shown in the study. First, the reliability of observational studies is often difficult to evaluate due to their non-randomized design. Therefore, to maintain the quality of the current network meta-analysis, only high-quality studies with a parallel randomized design were included, by the Oxford evidence level system. Maintenance of quality reduced the number of eligible studies included in the analysis. Smaller study numbers increase the potential for publication bias and small-study effects, especially in the case of the PBL network where the funnel plot and Egger’s test revealed a dubious publication bias. Moreover, although significant associations were found between mean BMI and TRF and between nature of surgery and IBL, the numbers of eligible studies were too small to allow a further subgroup analysis. In addition, the insufficiency of eligible studies also could lead to an imbalance between each group in the network. For example, numbers were very variable among groups with LO and PO being disproportionately small compared with IVLOW and IVLAR. Such an imbalance may impose ill effects on the results. Second, although SUCRA rankings have been widely used to give clinically significant results, cautious interpretation is required due to the minimum absolute difference between the highest rank and others [59]. Fourthly, network meta-analysis relies for its statistical stability and reliability on the uniform standards of similarity, homogeneity and consistency, and a range of statistical methods was used to test for heterogeneity and inconsistency. A significant local inconsistency was found in the PBL network by the node-split test but a satisfactory analysis of sensitivity could not be performed because of the small number of studies (only two studies with data on the COM group: Dong et al. [26] and Li et al. [27]). When further high-quality data becomes available, analysis can be extended but, up to that point, PBL network results should be interpreted with caution.

## Conclusion

The current network meta-analysis incorporated data from 33 studies with 3302 patients. In conclusion, there is no evidence that the use of TXA during spinal surgery is associated with a higher risk of embolism. The combination of intravenous and topical administration of TXA appears optimal for the reduction of perioperative bleeding and blood transfusion. An approach involving local infiltration is not effective for blood conservation during spinal surgery. Further studies are required to verify the current findings.

## Supplementary Material

Supplemental Material

## Data Availability

The data that support the findings of this study are available from the corresponding author upon reasonable request.

## Abbreviations

BMI: body mass index
CI: confidence interval
COM: combined use (intravenous plus topical)
DIC: deviance information criterion
dDIC: differences between each pair of DICs
HBC: change in haemoglobin during the 24-hour postoperative period
IBL: intraoperative blood loss
IV: intravenous
IVLAR: high-dose intravenous (≥20 mg/kg or >1g)
IVLOW: low-dose intravenous (<20 mg/kg or ≤1 g)
IVMUL: multiple intravenous
LO: local infiltration
MCMC: Markov-chain Monte Carlo
PBL: postoperative blood loss
PLA: placebo
PLIF: posterior lumbar interbody fusion
PO: oral
PRISMA: Preferred Reporting Items for Systematic Reviews and Meta-Analyses
RR: risk ratio
SUCRA: the surface under the cumulative ranking analysis
TOPLAR: high-dose topical (>1.5 g)
TOPLOW: low-dose topical (≤1.5 g)
TPA: tissue plasminogen activator
TRF: perioperative blood transfusion rate
TXA: tranexamic acid
WMD/MD: weighted mean difference
VTE: venous thrombosis
