# Supplementary material for: Optimal administration strategies of tranexamic acid to minimize blood loss during spinal surgery: results of a Bayesian network meta-analysis
Source: Ann Med. 2022 Jul 21;54(1):2053–63. doi: 10.1080/07853890.2022.2101687 (PMC9307111; doi:10.1080/07853890.2022.2101687)
Supplement: Supplemental Material [file IANN_A_2101687_SM2053.pdf]

Supplementary Figure 1. PRISMA Flow Diagram.

Supplementary Figure 2. Funnel plots. (A) IBL. (B) PBL. (C) HBC. (D) TRF. (E) VTE. (1: PLA; 2: LO; 3: PO; 4: TOPLOW; 5: TOPLAR; 6: IVLOW; 7: IVLAR; 8: IVMUL; 9:COM.) IBL: intraoperative blood loss; PBL: postoperative blood loss; HBC: change in hemoglobin during the 24-hour postoperative period; TRF: perioperative blood transfusion rate; VTE: venous thrombosis; PLA: placebo; LO: local infiltration; PO: oral; TOPLOW: low-dose topical; TOPLAR: high-dose topical; IVLOW: low-dose intravenous; IVLAR: high-dose intravenous; IVMUL: multiple intravenous; COM: combined use (intravenous plus topical).

Supplementary Figure 3. The details of the Egger's tests. (A) IBL. (B) PBL. (C) HBC. (D) TRF. (E) VTE (Positive results were considered when $P < 0.05$). IBL: intraoperative blood loss; PBL: postoperative blood loss; HBC: change in hemoglobin during the 24-hour postoperative period; TRF: perioperative blood transfusion rate; VTE: venous thrombosis.

Supplementary Figure 4. Node-split inconsistency tests of each endpoint networks. (A) IBL. (B) PBL. (C) HBC. (D) TRF. (E) VTE (Significant local inconsistency were considered when $P < 0.05$). IBL: intraoperative blood loss; PBL: postoperative blood loss; HBC: change in hemoglobin during the 24-hour postoperative period; TRF: perioperative blood transfusion rate; VTE: venous thrombosis.

Supplementary Figure 5. Iteration convergence diagnoses. (A) IBL. (B) PBL. (C) HBC. (D) TRF. (E) VTE. (Based on the Gelman-Rubin-Brooks method, the closer the potential scale reduction factor is to 1, the better the iteration converged.) IBL: intraoperative blood loss; PBL: postoperative blood loss; HBC: change in hemoglobin during the 24-hour postoperative period; TRF: perioperative blood transfusion rate; VTE: venous thrombosis.

Supplementary Figure 6. The history plots of iteration traces. (A) IBL. (B) PBL. (C) HBC. (D) TRF. (E) VTE. IBL: intraoperative blood loss; PBL: postoperative blood loss; HBC: change in hemoglobin during the 24-hour postoperative period; TRF: perioperative blood transfusion rate; VTE: venous thrombosis.

Supplementary Figure 7. Detailed results of SUCRA ranking. (A) IBL. (B) PBL. (C) HBC. (D) TRF. (E) VTE. IBL: intraoperative blood loss; PBL: postoperative blood

For Peer Review Only

loss; HBC: change in hemoglobin during the 24-hour postoperative period; TRF: perioperative blood transfusion rate; VTE: venous thrombosis.

Supplementary Table 1. Baseline Characteristics of included Studies.

Supplementary Table 2. Methodological quality and risk of bias evaluation of included study.

Supplementary Table 3. Global inconsistency and heterogeneity of each endpoint networks.

Supplementary Table 4. The detailed results of network meta-regression analysis.

For Peer Review Only

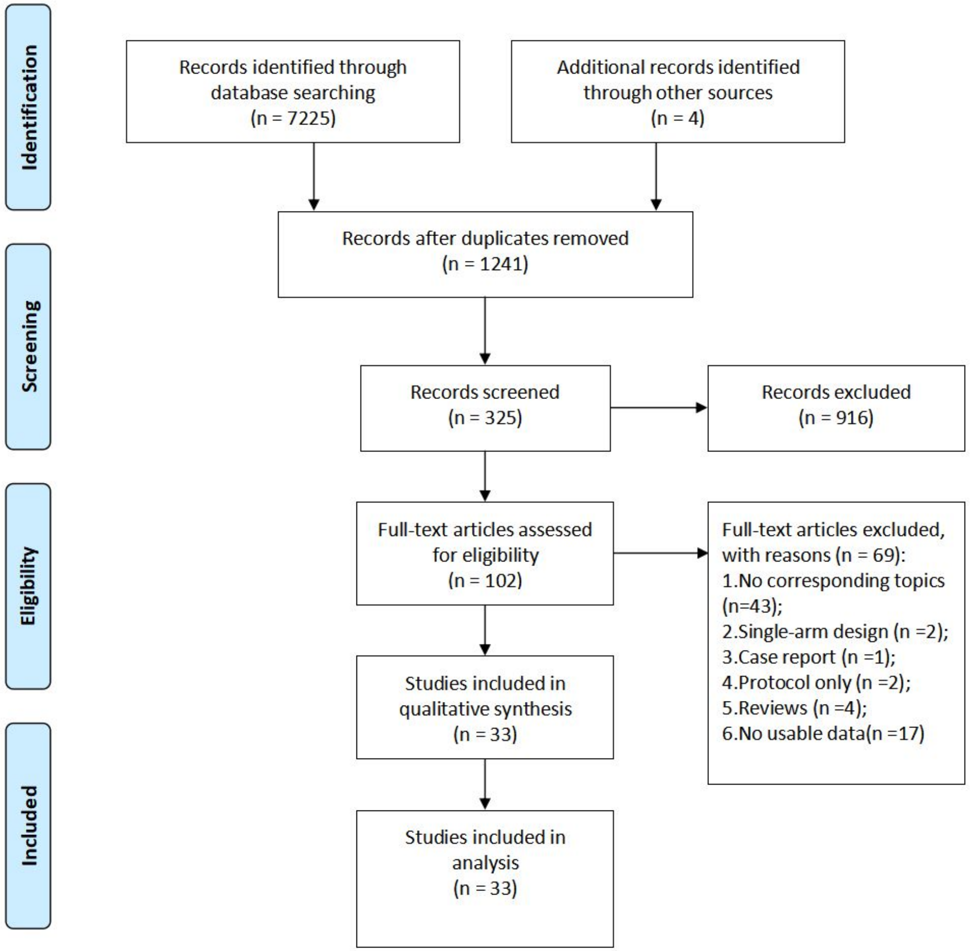

ew Only

Supplementary Figure 1. PRISMA Flow Diagram.

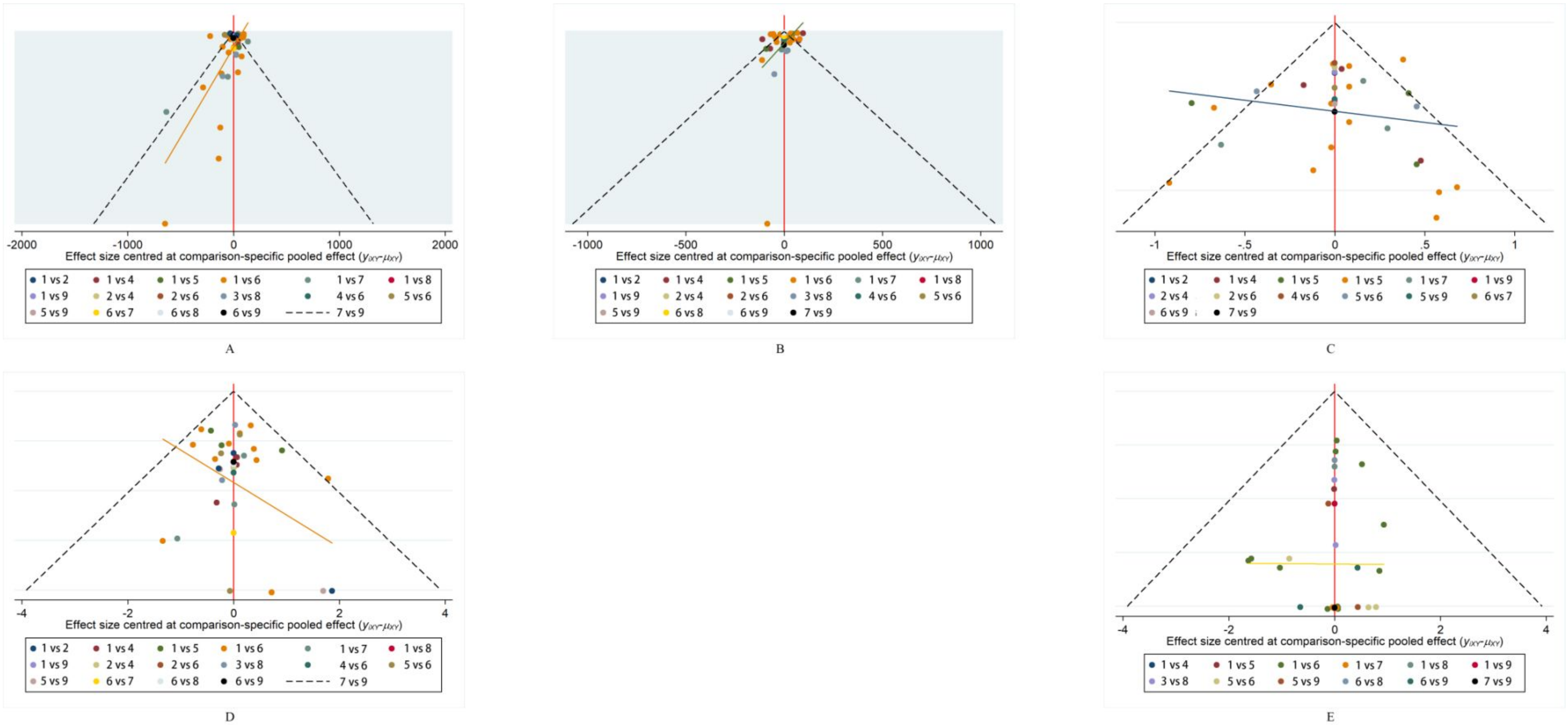

Supplementary Figure 2. Funnel plots. (A) IBL. (B) PBL. (C) HBC. (D) TRF. (E) VTE. (1: PLA; 2: LO; 3: PO; 4: TOPLOW; 5: TOPLAR; 6: IVLOW; 7: IVLAR; 8: IVMUL; 9:COM.) IBL: intraoperative blood loss; PBL: postoperative blood loss; HBC: change in hemoglobin during the 24-hour postoperative period; TRF: perioperative blood transfusion rate; VTE: venous thrombosis; PLA: placebo; LO: local infiltration; PO: oral; TOPLOW: low-dose topical; TOPLAR: high-dose topical; IVLOW: low-dose intravenous; IVLAR: high-dose intravenous; IVMUL: multiple intravenous; COM: combined use (intravenous plus topical).

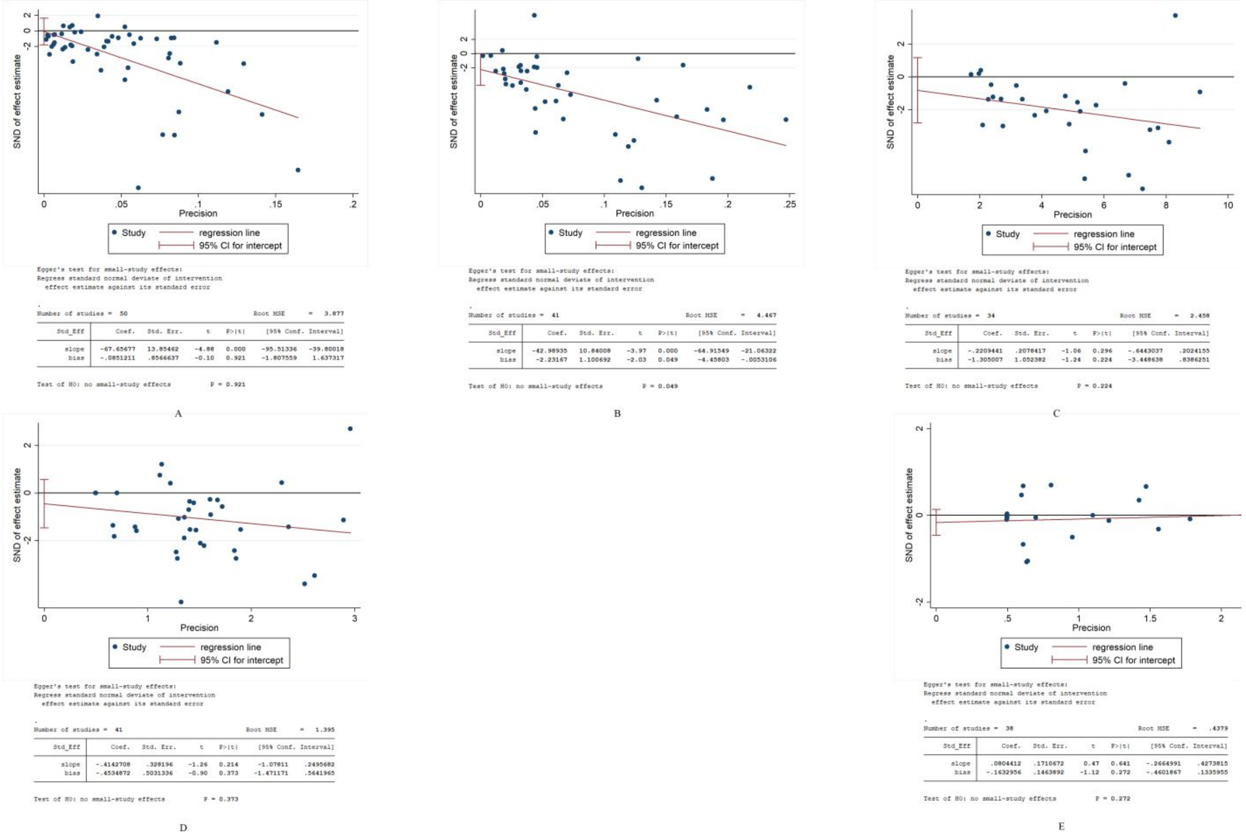

Supplementary Figure 3. The details of the Egger's tests. (A) IBL. (B) PBL. (C) HBC. (D) TRF. (E) VTE. (Positive results were considered when P < 0.05) IBL: intraoperative blood loss; PBL: postoperative blood loss; HBC: change in hemoglobin during the 24-hour postoperative period; TRF: perioperative blood transfusion rate; VTE: venous thrombosis.

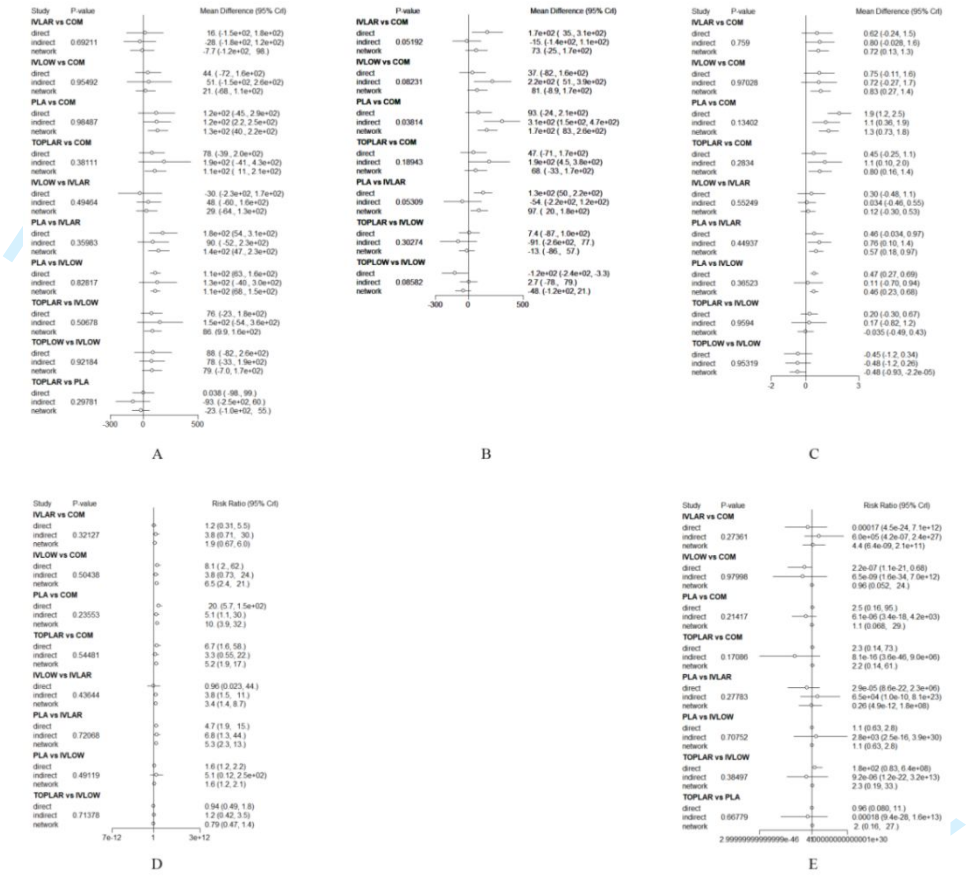

Supplementary Figure 4. Node-split inconsistency tests of each endpoint networks. (A) IBL. (B) PBL. (C) HBC. (D) TRF. (E) VTE (Significant local inconsistency were considered when P < 0.05). IBL: intraoperative blood loss; PBL: postoperative blood loss; HBC: change in hemoglobin during the 24-hour postoperative period; TRF: perioperative blood transfusion rate; VTE: venous thrombosis.

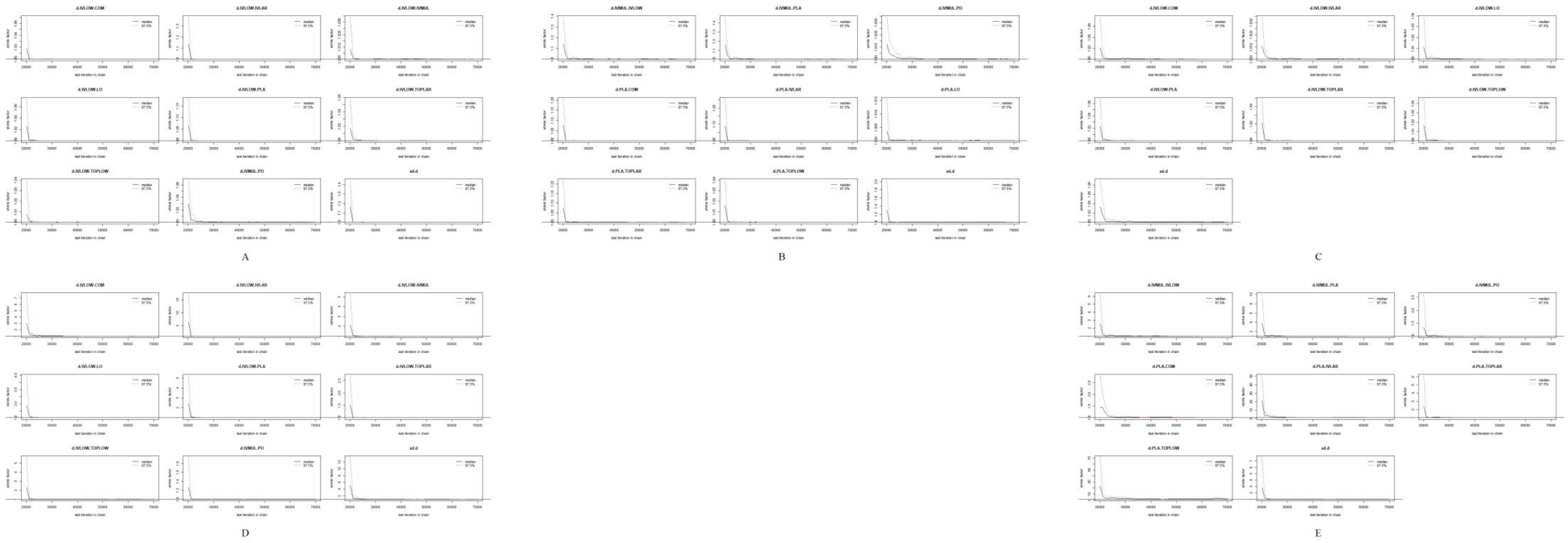

Supplementary Figure 5. Iteration convergence diagnoses. (A) IBL. (B) PBL. (C) HBC. (D) TRF. (E) VTE (Based on the Gelman-Rubin-Brooks method, the closer the potential scale reduction factor is to 1, the better the iteration converged). IBL: intraoperative blood loss; PBL: postoperative blood loss; HBC: change in hemoglobin during the 24-hour postoperative period; TRF: perioperative blood transfusion rate; VTE: venous thrombosis.

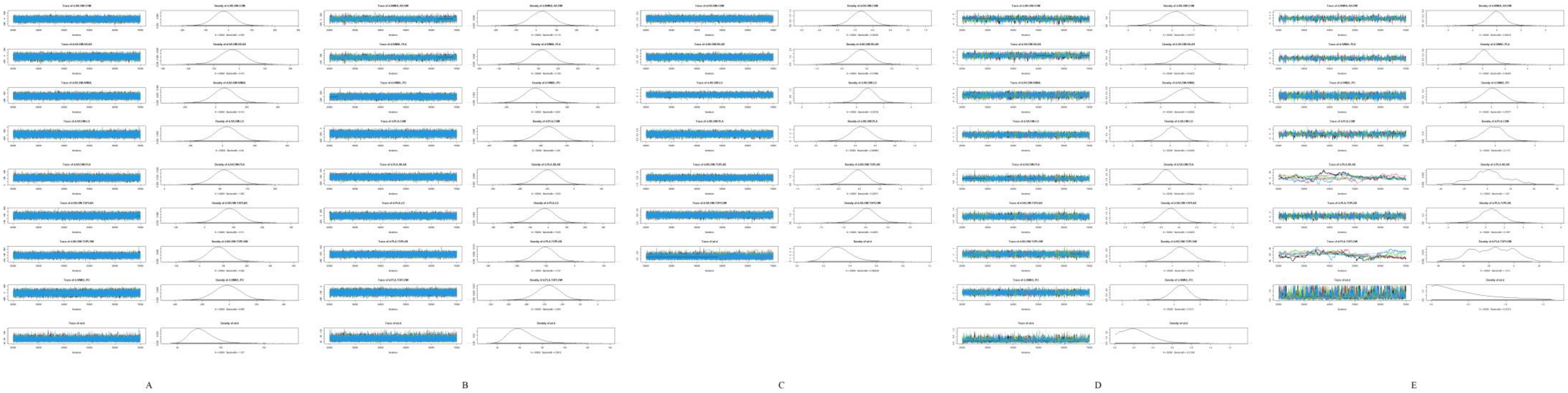

Supplementary Figure 6. The history plots of iteration traces. (A) IBL. (B) PBL. (C) HBC. (D) TRF. (E) VTE. IBL: intraoperative blood loss; PBL: postoperative blood loss; HBC: change in hemoglobin during the 24-hour postoperative period; TRF: perioperative blood transfusion rate; VTE: venous thrombosis.

Review Only

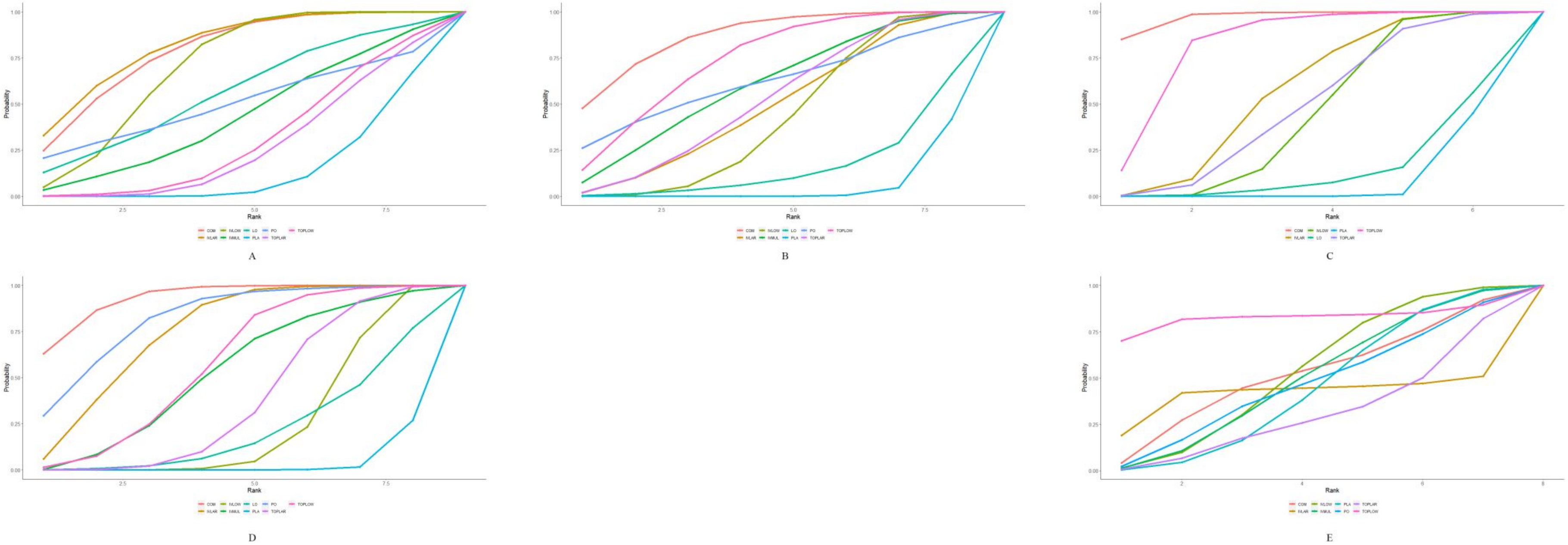

Supplementary Figure 7. Detailed results of SUCRA ranking. (A) IBL. (B) PBL. (C) HBC. (D) TRF. (E) VTE. IBL: intraoperative blood loss; PBL: postoperative blood loss; HBC: change in hemoglobin during the 24-hour postoperative period; TRF: perioperative blood transfusion rate; VTE: venous thrombosis.

Supplementary Table 1. Baseline Characteristics of included Studies

Author	No.	Year	Number of patients	Mean age	Percentage of Male (%)	Diagnosis	Surgery Type	Intervention I	Intervention II	Intervention III	Intervention IV
Carabini LM et. al.	1	2018	61	66.48	31.15	NL（exclude spinal tumor）	Multilevel Spine Fusion Surgery	Placebo	Low dose intravenous use of TXA	NA	NA
Neilipovitz DT et. al.	2	2001	40	13.92	42.50	Adloscent Scoliosis	Posterior spinal fusion	Placebo	Low dose intravenous use of TXA	NA	NA
Peters A et. al.	3	2015	32	53.09	NR	Adult spinal deformity	Posterior spinal fusion	Low dose intravenous use of TXA	Placebo	NA	NA
Dong Y et. al.	4	2021	80	14.20	32.5	Adolescent idiopathic scoliosis	Posterior spinal fusion	Combination of topical and intravenous use of TXA	High dose intravenous use of TXA	NA	NA
Li J et. al.	5	2020	280	65.79	35.36	2-level degenerative lumbar spine disease	2-level lumbar fusion	Combination of topical and intravenous use of TXA	Low dose intravenous use of TXA	High dose topical use of TXA	Placebo
Wang X et. al.	6	2019	181	45.54	57.46	Thoracolumbar Fracture	Internal fixation surgery	Low dose intravenous use of TXA	High dose topical use of TXA	Combination of topical and intravenous use of TXA	NA
Elwatidy S et. al.	7	2008	64	50.66	60.94	Degenerative disease and spinal tumor	Discectomy, laminectomy and segmental decompression	High dose intravenous use of TXA	Placebo	NA	NA
Farrokhi MR et. al.	8	2011	76	48.45	23.68	NR	Posterior spinal fusion	Placebo	Low dose intravenous use of TXA	NA	NA
Mu X et. al.	9	2019	126	52.90	57.14	Lumbar degenerative disease	Posterior spinal fusion	Low dose intravenous use of TXA	High dose topical use of TXA	Placebo	NA

For Peer Review Only

Yu CC et. al.	10	2019	83	62.55	40.96	NL（exclude spinal tumor）	Posterior spinal fusion	Multiple intravenous use of TXA	Oral use of TXA	NA	NA
Yu CC et. al.	11	2021	261	62.57	45.98	NL（exclude spinal tumor）	Posterior spinal fusion	Multiple intravenous use of TXA	Oral use of TXA	NA	NA
Lloyd M Halpern 1	12	2021	90	15.33	21.11	Adolescent idiopathic scoliosis	Posterior spinal fusion	Placebo	Low dose intravenous use of TXA	NA	NA
Sudprasert W et. al.	13	2018	57	51.75	61.40	Thoracolumbar spine trauma	Posterior spinal fusion	Low dose topical use of TXA	Placebo	NA	NA
Basavaraj K et. al.	14	2017	147	54.53	31.97	NR	Thoracicspine fixation	Low dose intravenous use of TXA	Placebo	NA	NA
Elmose S 1	15	2019	233	50.00	58.37	Symptomatic, MRI-verified lumbar spinal stenosis or disc herniations	Minor lumbar spine surgery (Open, Microscopic and Minimally invasive)	Low dose intravenous use of TXA	Placebo	NA	NA
Nagabhushan RM 1	16	2017	50	50.66	40.00	NR	Posterior spinal fusion	Low dose intravenous use of TXA	Placebo	NA	NA
Colomina MJ et. al.	17	2017	95	54.69	29.47	NL（exclude spinal tumor）	NL	Low dose intravenous use of TXA	Placebo	NA	NA
Arun-Kumar V et. al.	18	2021	104	50.25	47.12	Degenerative grade 1 or 2 spondylolisthesis	Single or dual level lumbar fixation with interbody fusions	Placebo	Local infiltration use of TXA	Low dose intravenous use of TXA	Low dose topical use of TXA
Kim KT et. al.	19	2017	72	63.17	51.39	Symptomatic lumbar spinal stenosis with borderline to grade 1 spondylolisthesis	Posterior lumbar interbody fusion	Placebo	Low dose intravenous use of TXA	Low dose intravenous use of TXA	NA
Shen J et. al.	20	2021	76	39.15	61.84	Thoracolumbar burst fracture	Posterior internal fxation	Low dose topical use of TXA	Placebo	NA	NA

For Peer Review Only

Seddighi A et. al.	21	2017	40	46.78	30.00	Degenerative disease	NL	Low dose intravenous use of TXA	Placebo	NA	NA
Wang W et. al.	22	2018	80	41.87	53.75	Thoracolumbar fracture dislocation	Transforaminal interbody fusion	Low dose intravenous use of TXA	Placebo	NA	NA
He B et. al.	23	2020	40	57.93	47.50	Degenerative disease	Posterior lumbar interbody fusion	Low dose intravenous use of TXA	Placebo	NA	NA
Hasan MS et. al.	24	2021	166	14.35	13.25	Adolescent Idiopathic Scoliosis	Posterior spinal fusion	High dose intravenous use of TXA	Low dose intravenous use of TXA	NA	NA
Goobie SM et. al.	25	2018	111	14.80	20.72	Adolescent Idiopathic Scoliosis	Posterior spinal fusion	Placebo	High dose intravenous use of TXA	NA	NA
Xu D et. al.	26	2020	60	50.10	45.00	Spondylolisthesis, spondylolysis, severe spinal instability or large disc herniation	NL	Placebo	Low dose topical use of TXA	NA	NA
Sethna NF et. al.	27	2005	44	13.79	68.18	Pediatric Scoliosis	Posterior or anterior-posterior spinal fusion	High dose intravenous use of TXA	Placebo	NA	NA
Wong J et. al.	28	2008	147	53.38	31.97	NL（exclude spinal tumor）	Posterior spinal fusion	Low dose intravenous use of TXA	Placebo	NA	NA
Tsutsumimoto T et. al.	29	2011	40	66.90	77.50	Compressive myelopathy	Cervical laminoplasty	Placebo	Low dose intravenous use of TXA	NA	NA
Shi H et. al.	30	2017	96	54.77	48.96	Lumbar stenosis or spondylolisthesis	Posterior spinal fixation or fusion	High dose intravenous use of TXA	Placebo	NA	NA
Wang Q et. al.	31	2013	60	61.55	56.67	Degenerative lumbar instability	Posterior spinal fusion	Placebo	Low dose intravenous use of TXA	NA	NA
Zhu X et. al.	32	2020	150	55.60	41.33	Degenerative disease	Posterior spinal fusion	Placebo	Low dose intravenous use of TXA	Multiple intravenous use of TXA	NA

For Peer Review Only

Liang J et. al.	33	2016	60	52.83	55.00	Degenerative disease	Posterior lumbar decompression and fusion	High dose topical use of TXA	Placebo	NA	NA

NA: Not applicable; NR: Not reported; NL: Not limited.

For Peer Review Only

Supplementary Table 2. Methodological quality and risk of bias evaluation of included study.

Author	No.	Sequence generation	Allocation concealment	Blinding	Incomplete outcome data	Selective outcome reporting	Other source of bias
Carabini LM et. al.	1	L	U	L	L	L	U
Neilipovitz DT et. al.	2	U	L	L	L	L	U
Peters A et. al.	3	L	L	L	L	L	U
Dong Y et. al.	4	L	L	L	L	L	U
Li J et. al.	5	U	U	U	L	L	U
Wang X et. al.	6	L	U	U	L	L	U
Elwatidy S et. al.	7	L	L	L	L	L	U
Farrokhi MR et. al.	8	L	U	L	L	L	U
Mu X et. al.	9	L	U	U	L	L	U
Yu CC et. al.	10	L	U	L	L	L	U
Yu CC et. al.	11	L	U	L	L	L	U
Lloyd M Halpern 1	12	U	L	U	L	L	L
Sudprasert W et. al.	13	L	L	L	L	L	U
Basavaraj K et. al.	14	L	U	L	L	L	U
Elmose S 1	15	L	U	L	L	L	U
Nagabhushan RM 1	16	L	U	L	L	L	U
Colomina MJ et. al.	17	L	U	L	L	L	U
Arun-Kumar V et. al.	18	U	U	U	L	L	U
Kim KT et. al.	19	L	U	L	L	L	U
Shen J et. al.	20	L	L	L	L	L	U
Seddighi A et. al.	21	U	U	U	U	L	U
Wang W et. al.	22	L	L	L	L	L	U
He B et. al.	23	L	L	L	L	L	U

For Peer Review Only

Hasan MS et. al.	24	L	L	L	L	L	U
Goobie SM et. al.	25	L	L	L	L	L	U
Xu D et. al.	26	L	U	L	L	L	U
Sethna NF et. al.	27	L	U	L	U	L	U
Wong J et. al.	28	L	L	L	L	L	U
Tsutsumimoto T et. al.	29	L	U	L	L	L	U
Shi H et. al.	30	L	L	L	L	L	U
Wang Q et. al.	31	L	U	L	L	L	U
Zhu X et. al.	32	L	L	L	L	L	U
Liang J et. al.	33	L	L	L	L	L	U

L: low risk of bias. U: unclear risk of bias. H: high risk of bias.

For Peer Review Only

Supplementary Table 3. Global inconsistency and heterogeneity of each endpoint networks.

Network	Consistency model			Inconsistency model			dDIC
	DIC	Global heterogeneity, I2	Data points	DIC	Global heterogeneity, I2	Data points	
IBL	137.87204	0.9 %	75	139.14546	0.2 %	75	1.27342
PBL	113.38513	0.0 %	60	113.44070	0.0 %	60	0.05557
HBC	90.31420	4.0 %	49	91.16916	5.0 %	49	0.85496
TRF	80.45547	0.0 %	57	86.35271	0.0 %	57	5.89724
VTE	48.11085	0.0 %	59	46.12896	0.0 %	59	1.98189

DIC:deviance information criterion; dDIC:the difference between each pair of DICs. IBL: intraoperative blood loss; PBL: postoperative blood loss; HBC: change in hemoglobin during the 24-hour postoperative period; TRF: perioperative blood transfusion rate; VTE: venous thrombosis.

For Peer Review Only

Supplementary Table 4. The detailed results of network meta-regression analysis.

Covariate	Publication year of included studies			Mean BMI of participants			Mean age of participants			Disease and surgery type of participants		
	mean	SD	95% CI	mean	SD	95% CI	mean	SD	95% CI	mean	SD	95% CI
β (IBL)	50.66	63.29	(-68.75, 179.10)	-81.98	61.23	（-203.03, 38.69)	83.97	52.34	(-16.587, 189.24)	113.94	40.18	(32.27， 190.86) *
β (PBL)	55.16	32.08	(-6.79， 121.02)	-4.94	49.13	(-101.66,92.50)	-15.26	32.86	(-79.95, 50.21)	24.34	36.52	(-46.30, 97.65)
β (HBC)	-0.16	0.23	(-0.62, 0.29)	-0.13	0.30	(-0.74, 0.46)	0.96	1.99	(-3.60, 4.74)	-0.12	0.25	(-0.60, 0.37)
β (TRF)	-0.57	0.31	(-1.18, 0.05)	1.52	0.62	(0.34, 2.76) *	0.28	0.56	(-0.72, 1.45)	-0.29	0.49	(-1.22, 0.72)
β (VTE)	0.64	1.42	(-1.92, 3.84)	0.04	0.72	(-1.37, 1.56)	-0.73	1.03	（-3.12， 1.05）	-0.1	0.88	(-1.90, 1.62)

β : The shared regression coefficients; *: Significant interaction. IBL: intraoperative blood loss; PBL: postoperative blood loss; HBC: change in hemoglobin during the 24-hour postoperative period; TRF: perioperative blood transfusion rate; VTE: venous thrombosis.

For Peer Review Only
